# Moderately hypofractionated prostate-only versus whole-pelvis radiotherapy for high-risk prostate cancer: A retrospective real-world single-center cohort study

**DOI:** 10.1016/j.ctro.2024.100846

**Published:** 2024-08-21

**Authors:** Jenny Kahlmeter Brandell, Antonis Valachis, Henrik Ugge, Daniel Smith, Bengt Johansson

**Affiliations:** a Department of Oncology, Faculty of Medicine and Health, Örebro University Hospital, Örebro University, Örebro, Sweden; b Department of Urology, Faculty of Medicine and Health, Örebro University Hospital, Örebro University, Örebro, Sweden; c Clinical Epidemiology and Biostatistics, School of Medical Sciences, Örebro University, 702 81 Örebro, Sweden

**Keywords:** Prostate cancer, Radiotherapy, Pelvis, Radiation dose hypofractionation, Quality of life

## Abstract

**Background:**

The benefit of prophylactic whole pelvis radiation therapy (WPRT) in prostate cancer has been debated for decades, with evidence based mainly on conventional fractionation targeting pelvic nodes.

**Aim:**

This retrospective cohort study aimed to explore the impact of adding moderately hypofractionated pelvic radiotherapy to prostate-only irradiation (PORT) on prognosis, toxicity, and quality of life in real-world settings.

**Materials and methods:**

Patients with high-risk and conventionally staged prostate cancer (cT1-3N0M0) treated with moderately hypofractionated WPRT or PORT, using external beam radiotherapy alone or combined with high-dose-rate brachytherapy, at Örebro University Hospital between 2008 and 2021 were identified. Biochemical failure-free survival (BFFS), metastasis-free survival (MFS), prostate cancer-specific survival (PCSS), and overall survival (OS) were compared using Kaplan-Meier method and Cox proportional hazards. Toxicity and quality of life measures were also analysed.

**Results:**

Among 516 patients (227 PORT, 289 WPRT), 5-year BFFS rates were 77 % (PORT) and 74 % (WPRT), adjusted HR=1.50 (95 % CI=0.88–2.55). No significant differences were found in MFS, PCSS, or OS in main analyses. WPRT was associated with a higher risk of acute grade ≥ 2 and 3 genitourinary toxicities whereas no differences in late toxicities or quality of life between PORT and WPRT were observed.

**Conclusion:**

We found no significant differences in oncological outcomes or quality of life when comparing moderately hypofractionated PORT to WPRT. Some differences in toxicity patterns were observed. Despite caveats related to study design, our findings support the need for further research on WPRT’s impact on treatment-related and patient-reported outcomes.

## Highlights

• Moderately hypofractionated WPRT did not improve oncological outcomes.
• No observed difference in QoL, GI, or sexual toxicity between PORT and WPRT.
• WPRT increased the risk of severe acute GU toxicity.
• Further studies on hypofractionated WPRT are needed.

## 1 Introduction

External beam radiotherapy (EBRT), alone or with high-dose-rate brachytherapy (HDR-BT), along with androgen deprivation therapy (ADT), is a standard treatment for high-risk prostate cancer [1], [2]. Prophylactic irradiation of pelvic lymph nodes (LN), commonly referred to as whole pelvis radiation therapy (WPRT), targets hidden microscopic LN disease. However, its survival benefit remains debated [3], [4], [5], [6], [7], [8], [9], [10], [11] and the recommended approaches vary [1], [2].

The GETUG-01 trial [3], [4] found no biochemical failure-free survival (BFFS) benefit when adding pelvic irradiation to prostate-only radiotherapy (PORT). The Radiation Therapy Oncology Group (RTOG) 9413 study [5], [6] showed improved progression-free survival (PFS) with WPRT [5], but this was less evident over time [6]. In GETUG-01 [3] 55 % of the patients had LN-risk < 15 %, mini-pelvic fields were utilized, and hormonal therapy was not controlled for. In RTOG 9413 [5] all patients had LN risk > 15 %, upper LN-border was L5-S1, and all patients received 4 months of ADT (either adjuvant or neoadjuvant). In both studies the prostate dose was suboptimal when compared to contemporary dose-escalated radiotherapy [12]. In 2021 The POP-RT trial [7] demonstrated improved BFFS and metastasis-free survival (MFS) with WPRT over PORT, using larger LN-volumes and two years of ADT for high-risk prostate cancer with LN risk ≥ 20 %, as per the Roach formula [13]. Andruska et al. [8] showed improved overall survival (OS) with WPRT for patients with high LN risk in a large retrospective study, whereas Amini et al. [11], found no survival advantage.

Current evidence on WPRT in curative prostate cancer treatment is mainly based on conventional fractionation to pelvic nodes [14]. Besides, most real-world evidence on WPRT has been focused on prognosis [8], [10], [11], with limited data on toxicity and quality of life (QoL).

At Örebro University Hospital, moderately hypofractionated EBRT has been employed for both PORT and WPRT since 2008. The aim of the present retrospective cohort study was to investigate the impact of adding moderately hypofractionated pelvic radiotherapy to prostate-only irradiation in terms of prognosis, toxicity, and QoL in a real-world context of conventionally staged patients with high-risk prostate cancer.

## 2 Material and methods

This study is reported in accordance with the European Society for Medical Oncology Guidelines for Reporting Oncology Work (ESMO-GROW) [15].

### 2.1 Study cohort

A source population of 552 patients with high-risk prostate cancer, cT1-3N0M0, consecutively treated with moderately hypofractionated EBRT, alone or with HDR-BT, at Örebro University Hospital between 2008 and 2021 were identified from a prospectively maintained institutional database. Eligible patients had histologically confirmed high-risk prostate cancer without regional or distant metastases on conventional imaging. High-risk was defined as cT3, Gleason score ≥ 8 or PSA≥20 ng/mL, consistent with National Comprehensive Cancer Network (NCCN) guidelines [2]. All patients received an information letter at the study start with the option to withdraw their participation.

We excluded 16 patients due to long-term hormonal treatment and PSA progression pre-radiotherapy, three for not completing the treatment, two misclassified into the wrong risk group, one with missing follow-up data and 14 who opted out, leaving 516 patients eligible for analysis.

### 2.2 Treatment planning

Patients underwent either PORT or WPRT. The PORT group received EBRT at 66 Gy over 22 fractions, or a 14.5 Gy HDR-BT boost with 42 Gy EBRT in 14 fractions. WPRT involved 42 Gy in 14 fractions to the whole pelvis with a sequential boost to the prostate to 66 Gy, or a 14.5 Gy HDR-BT boost. EBRT was delivered with 3-dimensional conformal technique (3DCRT) from 2008 to 2013 and volumetric modulated arc therapy (VMAT) from 2014 onwards. Moderate hypofractionation (3 Gy/fraction, thrice weekly) was used for both prostate and LN irradiation. EBRT was image-guided, using either fiducial-based or bone-based methods.

The prostate's clinical target volume (CTV) was delineated based on CT for EBRT and ultrasound for HDR-BT. The planning target volume (PTV) for EBRT included a 7 mm margin with fiducial markers or a 10–15 mm margin for bone-based set-up, in accordance with regional guidelines at the time. For HDR-BT, PTV equalled CTV. Pelvic LN CTV varied over time, with a generally larger volume from 2014 onward, with upper limit at L5/S1, encompassing external iliac nodes. Obturator nodes were included in all patients, while presacral and posterior mesorectal nodes were not included. Pelvic LN PTV=CTV+5–7 mm. Organs at risk (bowel-bag, rectum, bladder, and femoral heads) were delineated according to local guidelines for EBRT. Urethra, rectal wall, and rectal mucosa was delineated 5 mm beyond CTV for HDR-BT. Dose constraints for EBRT were modified from QUANTEC [16] and Fiorino et al [17]. The HDR-BT method and dose constraints are published elsewhere [18].

### 2.3 Follow-up strategy

Patients were monitored per institutional protocols, with a clinical visit at the completion of radiotherapy, a three-week oncology nurse contact, and standardized questionnaires every six months for three years, then annually for ten years. At each follow-up, PSA levels were monitored and genitourinary (GU), gastrointestinal (GI), erectile dysfunction (ED) and sexual life (SL) toxicity were assessed using a modified RTOG grading scale [19] (Appendix A, Table A.1). The patient-reported Life-Satisfaction Questionnaire-11 (Lisat-11) [20], [21] was evaluated at baseline, three, five and ten years. Radiological assessments occurred only when clinically indicated.

### 2.4 Data collection

Data on patient- and tumour characteristics, treatment parameters, effects, toxicity and QoL were extracted from the database. Tumour characteristics, PSA-values, date for biochemical recurrence and metastatic disease, time- and cause of death, and radiotherapy parameters were confirmed and augmented by reviewing patient records and radiation systems.

### 2.5 Definitions and outcomes

Primary endpoint was BFFS, with biochemical failure defined as an absolute increase in serum PSA≥2 ng/ml above nadir, as per the Phoenix criteria [22].

Secondary endpoints included MFS, prostate cancer-specific survival (PCSS), OS, acute and late GU-, GI-, and sexual toxicities and long-term QoL. MFS was defined as freedom from regional and distant metastasis. Cause of death was obtained from patient records. If unknown, patients with metastatic Castration-Resistant Prostate Cancer (mCRPC) were recorded as prostate cancer deaths. Patients free from recurrence or with stable hormone-sensitive disease were classified as dying from other causes.

Toxicities of any grade were included in the analyses, with acute toxicities occurring within 3 months post-radiation and late toxicities occurring thereafter. LiSat-11 [20], [21] evaluates overall and specific life satisfaction in 11 domains. However, only total scores were available and analysed.

Patients without biochemical failure were censored at their last PSA test (for BFFS), metastasis-free patients at the last review of patient records or death (for MFS), and surviving patients at the last population registry cross-reference (for PCSS and OS). Follow-up started at radiotherapy completion.

LN risk was determined using the Roach formula [13].

This study was approved by the Swedish Ethics Review Board (reference number 2012–293, 2022–04694-02, 2024–00579-02).

### 2.6 Statistical analysis

Survival rates for time-to-event endpoints were estimated using the Kaplan-Meier method and compared via a two-sided log-rank test. Cox proportional hazards regression models estimated crude and adjusted hazard ratios (HRs) and 95 % Confidence Intervals (95 % CI). The multivariable model included radiation volume (PORT versus WPRT), age, year of treatment, T-stage, International Society of Urologic Pathologists (ISUP)-grade, PSA, HDR-BT use, and type of hormonal treatment. Simple imputation addressed missing ISUP-grade values (5 patients), while other variables were complete.

As a sensitivity analysis, cumulative incidence for MFS and PCSS was estimated with death and death due to other cause, respectively, as competing events and compared using Gray’s test. Subdistribution hazards were estimated with Fine-Gray regression including the same covariates as the Cox model.

Toxicity grades were reported at baseline, three weeks, one year, three years, five years and as the cumulative worst toxicity grade from six months to three years. The proportion of toxicities (Grade 0–1 versus ≥ 2 for all toxicities and Grade 0–2 versus ≥ 3 for GU toxicity) was estimated at three weeks and cumulatively at three years. Odds Ratios (ORs) and 95 % CI were calculated using a binomial linear regression model, including radiation volume, baseline toxicity grade (if available), age, radiation technique (3DCRT versus VMAT), prostate volume, and HDR-BT use as covariates.

The total LiSat-11 score was compared at three and five years. ORs with 95 % CI and adjusted mean scores were estimated using an ordinal regression model, including radiation volume, baseline score, age, radiation technique, and HDR-BT use.

A p-value of ≤ 0.05 was considered statistically significant. All analyses were performed in R version 4.3.2. GraphPad Prism version 10.2.3 was used for construction of toxicity and QoL plots.

## 3 Results

### 3.1 Study population

The study included 516 patients, 227 of whom received PORT and 289 underwent WPRT. Due to changes in national guidelines, the recommended treatment varied over time. From 2008 to 2015, WPRT was primarily administered, transitioning to PORT from 2015 to 2021 (Appendix A, Fig. A.1).

Patient and treatment characteristics are summarized in Table 1. More patients in the WPRT group were classified as clinical stage T3 and met more than one high-risk factor (54 % in WPRT versus 32 % in PORT), whereas a higher proportion of patients in the PORT group were ISUP grade 5. Median nodal risk was similar between the groups (33 %). More patients in the PORT group received HDR-BT boost and EBRT with VMAT technique. Most patients received neoadjuvant and adjuvant hormone therapy, predominantly first-generation antiandrogens, for two years after completion of radiotherapy.

**Table 1:** Patient and treatment characteristics.

		**Radiation volume**		
**Variable**	**Total,** N=516*^1^*	**PORT**, N=227*^1^*	**WPRT**, N=289*^1^*	**p-value***^2^*
**Age at diagnosis (years)**	70 (67, 74)	71 (67, 75)	69 (67, 73)	0.049
**Year of diagnosis**	2014 (2011, 2017)	2018 (2016, 2019)	2012 (2010, 2014)	<0.001
**PSA (ng/mL)**	20 (10, 32)	17 (9, 29)	21 (10, 35)	0.047
**ISUP grade**				<0.001
ISUP grade ≤ 3	210 (41 %)	98 (43 %)	112 (39 %)	
ISUP grade 4	141 (27 %)	41 (18 %)	100 (35 %)	
ISUP grade 5	160 (31 %)	87 (38 %)	73 (25 %)	
Unknown	5 (1.0 %)	1 (0.4 %)	4 (1.4 %)	
**Clinical T-stage**				<0.001
1	123 (24 %)	68 (30 %)	55 (19 %)	
2	156 (30 %)	78 (34 %)	78 (27 %)	
3	237 (46 %)	81 (36 %)	156 (54 %)	
**Number of high-risk factors^3^**				<0.001
1	286 (56 %)	154 (68 %)	132 (46 %)	
2	170 (33 %)	57 (25 %)	113 (40 %)	
3	55 (11 %)	15 (6.6 %)	40 (14 %)	
Unknown	5	1	4	
**Nodal risk (%)^4^**	33 (24, 41)	33 (24, 41)	33 (25, 44)	0.2
Unknown	5	1	4	
**Nodal risk (stratified)^4^**				0.045
Low (<20 %)	75 (15 %)	43 (19 %)	32 (11 %)	
Medium (≥20 %, ≤ 40 %)	285 (56 %)	121 (54 %)	164 (58 %)	
High (>40 %)	151 (30 %)	62 (27 %)	89 (31 %)	
Unknown	5	1	4	
**Prostate volume (cc)**	40 (30, 52)	40 (30, 50)	40 (30, 53)	0.8
Unknown	16	3	13	
**Radiation technique**				<0.001
3DCRT	189 (37 %)	26 (11 %)	163 (56 %)	
VMAT	327 (63 %)	201 (89 %)	126 (44 %)	
**Fiducial**				<0.001
No	290 (56 %)	102 (45 %)	188 (65 %)	
Yes	226 (44 %)	125 (55 %)	101 (35 %)	
**Seminal vesicles included**				<0.001
No	53 (10 %)	46 (20 %)	7 (2.4 %)	
Yes	463 (90 %)	181 (80 %)	282 (98 %)	
**Brachytherapy boost**				<0.001
No	166 (32 %)	54 (24 %)	112 (39 %)	
Yes	350 (68 %)	173 (76 %)	177 (61 %)	
**Hormonal treatment**				<0.001
ADT or CAB	68 (13 %)	34 (15 %)	34 (12 %)	
Antiandrogen only	428 (83 %)	174 (77 %)	254 (88 %)	
None	20 (3.9 %)	19 (8.4 %)	1 (0.3 %)	
**Sequence of hormonal therapy**				<0.001
Neoadjuvant only	29 (5.6 %)	24 (11 %)	5 (1.7 %)	
Adjuvant only	7 (1.4 %)	1 (0.4 %)	6 (2.1 %)	
Neoadjuvant and adjuvant	460 (89 %)	183 (81 %)	277 (96 %)	
None	20 (3.9 %)	19 (8.4 %)	1 (0.3 %)	
**Duration of hormonal therapy (months)**	28 (27, 29)	29 (27, 30)	28 (27, 29)	0.9
Unknown	20	19	1	

^1^Data presented in n (%) or median (IQR). ^2^Wilcoxon rank sum test; Fisher's exact test; Pearson's Chi-squared test. ^3^Number of high-risk factors (PSA≥20, T3, ISUP grade 4–5). ^4^Nodal risk according to Roach formula [13].

Abbreviations: PORT, Prostate Only Radiotherapy; WPRT, Whole Pelvis Radiation Therapy; PSA, Prostate Specific Antigen; ISUP, International Society of Urologic Pathologists; 3DCRT, Three Dimensional (3D) Conformal Radiation Therapy; VMAT, Volumetric modulated arc therapy; ADT, Androgen Deprivation Therapy; CAB, Combined Androgen Blockade, i.e., first generation antiandrogen in combination with Gonadotropin-releasing hormone-agonist or antagonist.

### 3.2 Effectiveness of WPRT

**Fig. 1:**
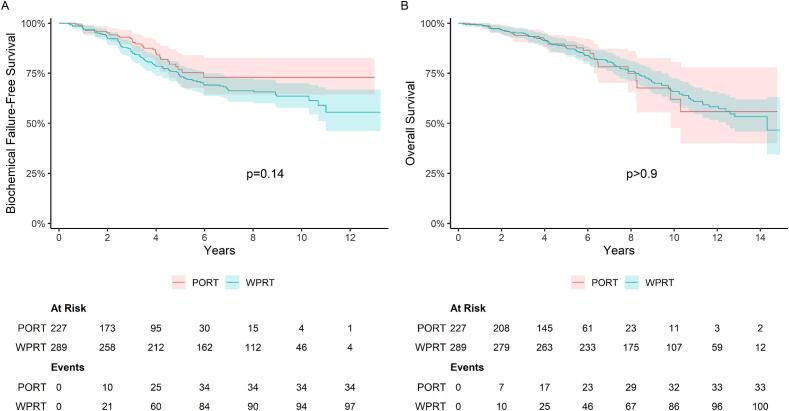
Biochemical failure free survival and Overall survival, PORT versus WPRT. Kaplan-Meier estimates of biochemical failure-free survival (A) and Overall survival (B). *Abbreviations:* PORT, Prostate Only Radiotherapy; WPRT, Whole Pelvis Radiation Therapy.

Median follow-up for BFFS was 46 months (IQR 24-59) for the PORT group and 83 months (IQR 47–115) for the WPRT group. A total of 131 biochemical failures were observed (34 in PORT, 97 in WPRT). The unadjusted 5-year BFFS rates were 77 % (95 % CI 70–85) for PORT and 74 % (95 % CI 69-79) for WPRT (Fig. 1). There was no significant association between radiation volume (PORT versus WPRT) and BFFS in the crude (HR=1.35, 95 % CI 0.90–2.01) or adjusted Cox regression model (aHR=1.50, 95 % CI 0.88–2.55; Table 2). Factors associated with prolonged BFFS included earlier year of treatment, lower T-stage, lower ISUP-grade, lower PSA, and the use of brachytherapy boost.

**Table 2:** Multivariate Cox regression analysis for biochemical failure-free survival within the whole study population.

**Characteristic**	**Event N**	**HR**^1^	**95 % CI**^1^	**p-value**
Radiation volume	131			
PORT		—	—	
WPRT		1.50	0.88, 2.55	0.14
Age	131	0.99	0.95, 1.03	0.6
Year of treatment	131	1.10	1.01, 1.19	0.021
T-stage	131			
1		—	—	
2		2.74	1.46, 5.16	0.002
3		3.48	1.90, 6.37	<0.001
ISUP-grade	131			
ISUP grade ≤ 3		—	—	
ISUP grade 4		1.81	1.13, 2.90	0.013
ISUP grade 5		2.48	1.55, 3.96	<0.001
PSA	131	1.01	1.00, 1.01	0.012
Brachytherapy boost	131			
No		—	—	
Yes		0.65	0.45, 0.95	0.024
Hormonal therapy	131			
None		—	—	
Antiandrogen only		0.76	0.23, 2.56	0.7
ADT or CAB		0.54	0.15, 2.00	0.4

^1^HR=Hazard Ratio, CI=Confidence Interval.

*Abbreviations:* PORT, Prostate Only Radiotherapy; WPRT, Whole Pelvis Radiation Therapy; ISUP, International Society of Urologic Pathologists; PSA, Prostate Specific Antigen; ADT, Androgen Deprivation Therapy; CAB, Combined Androgen Blockade, i.e., first generation antiandrogen in combination with Gonadotropin-releasing hormone-agonist or antagonist.

107 patients developed regional or distant metastasis (PORT=28, WPRT=79). Five-year MFS was 85 % (95 % CI 79–91) for PORT and 82 % (95 % CI 78–87) for WPRT (aHR=1.43, 95 % CI 0.78–2.61). Total number of deaths was 134, with 101 occurring in the WPRT group and 33 in the PORT group. Among these, 47 deaths in the WPRT group and 7 deaths in the PORT group were related to prostate cancer. The 5-year PCSS was 96 % (95 % CI 94–99) in the PORT group and 93 % (95 % CI 91–96) in the WPRT group. A higher PCSS rate was observed in the PORT compared to the WPRT group (HR=2.33, 95 % CI 1.03–5.27) though this difference was not statistically significant in the adjusted model (aHR=2.77, 95 % CI 0.99–7.79). The unadjusted 5-year OS was 89 % (95 % CI 84–94) for PORT versus 88 % (95 % CI 84–92) for WPRT (aHR=1.06, 95 % CI 0.61–1.83) (Fig. 1).

In sensitivity analyses for MFS and PCSS, where death and death due to other causes were considered as competing risk events, respectively, we found similar results as in main analyses for MFS (HR=1.49, 95 % CI 0.81–2.73; Appendix A, Table A.2) but a statistically significant worse PCSS in the WPRT group (HR=3.09 95 % CI 1.23–7.78; Appendix A, Table A.3).

### 3.3 Toxicity and quality of life

**Fig. 2:**
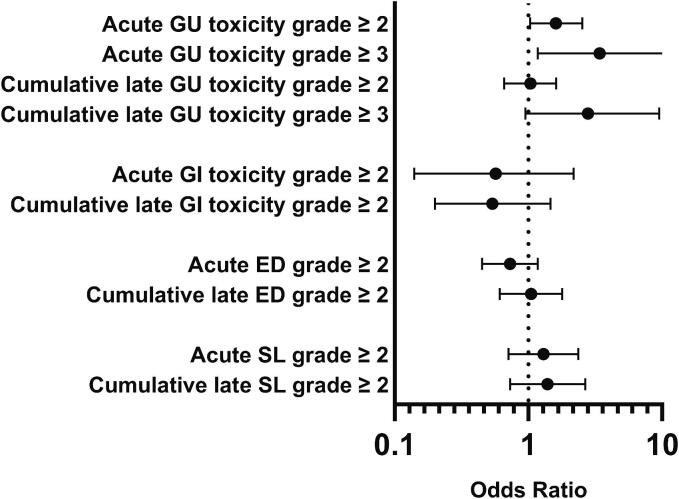
Association of acute and cumulative late toxicities with radiation volume. OR=Odds ratio (PORT:WPRT). Proportions of toxicity (Grade 0–1 versus ≥ 2 for all toxicities and Grade 0–2 versus ≥ 3 for GU toxicity) were estimated, and ORs calculated using a binomial regression model. The model included radiation volume, baseline toxicity grade*, age, radiation technique (3DCRT versus VMAT), prostate volume, and use of HDR-BT boost. * Not available and therefore not included in the model for GI-toxicity. *Abbreviations:* PORT, Prostate Only Radiotherapy; WPRT, Whole Pelvis Radiation Therapy; GU, genitourinary; GI, gastrointestinal; ED, erectile dysfunction; SL, sexual life; 3DCRT, Three Dimensional (3D) Conformal Radiation Therapy; VMAT, Volumetric modulated arc therapy.

Toxicity distributions are summarized in Appendix A, Fig. A.2. Baseline GI toxicity data were unavailable. Grade ≥ 3 GU toxicity was observed in 1.4 % (PORT) and 4.6 % (WPRT) at baseline, rising to 2.3 % versus 6.7 % at three weeks, and 2.5 % versus 7.4 % cumulatively over three years. No acute grade 3 GI toxicity occurred, with one late grade 3 GI toxicity in each group. Most patients in both groups had grade 1–2 ED and SL impairment at baseline, which increased during follow-up. Fig. 2 shows no significant association between radiation volume and acute or cumulative late grade 0–1 versus ≥ 2 toxicity for GI, ED or SL. However, WPRT increased the risk of acute grade ≥ 2 (OR=1.61, 95 % CI 1.03–2.52) and grade ≥ 3 (OR=3.41, 95 % CI 1.18 – 11.39) GU toxicity.

**Fig. 3:**
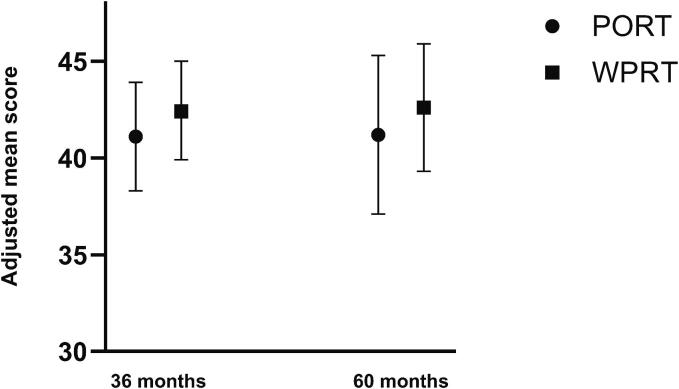
Adjusted mean scores of Quality-of-Life assessments for PORT versus WPRT at three and five years. Adjusted mean scores for Life-Satisfaction Questionnaire-11 at three and five years for PORT versus WPRT, estimated using an ordinal regression model, including radiation volume, baseline score, age, radiation technique, and use of Brachytherapy boost. *Abbreviations:* PORT, Prostate Only Radiotherapy; WPRT, Whole Pelvis Radiation Therapy.

Mean baseline score for LiSat-11 was 50.0 (SD 8.6) for PORT and 51.8 (SD 8.4) for WPRT. Adjusted mean scores at three and five years are shown in Fig. 3, indicating no difference between the groups (OR=0.89, 95 % CI 0.64–1.24 at three years; 0.86, 95 % CI 0.54–1.38 at five years).

## 4 Discussion

Our retrospective study found no statistically significant differences in BFFS, MFS, or OS among high-risk prostate cancer patients treated with PORT versus WPRT using moderately hypofractionated radiotherapy. For PCSS, we observed some indications favouring the PORT group, although this observation should be interpreted with caution due to the limited number of events. WPRT was associated with a higher risk of severe GU toxicities, while GI toxicities, sexual dysfunction, and QoL were similar between the groups.

The lack of treatment benefit from WPRT contradicts recent studies [7], [8], [9], [10]. Potential explanations related to patient selection, definition of pelvic nodal volume, patterns of hormonal therapy, and differences in study design, should be considered when comparing our results with others.

The characteristics of the selected patient population warrant consideration. With a median LN risk of 33 %, our study population is of considerably higher risk compared to the GETUG-01 [3] and RTOG 9413 [5] studies, though not as high as the POP-RT trial [7], which had a median LN risk of 38 %. Unlike POP-RT, which utilized PSMA-PET/CT for staging, we used conventional radiology. This methodological difference may have resulted in a higher proportion of patients with occult metastases in our high-risk cohort. Nevertheless, Andruska et al. [8] demonstrated an OS benefit with WPRT in conventionally staged patients with high LN risk.

Variations in pelvic nodal volume definitions may also contribute to differing results. According to current NRG guidelines [23] WPRT should encompass common iliac, external iliac- and presacral nodes; criteria not fully met in our study, where the LN coverage aligns more closely with the negative trials GETUG-01 [3] and RTOG 9413 [5]. Spratt et al. [24] demonstrated that LN failures frequently occur in common iliac nodes, superior to the upper border in our study. Additionally, differences in hormonal treatment may influence the results [7], [8], [24].

Furthermore, it is important to acknowledge the inherent risk of bias associated with the observational nature of our study. Although treatment decision largely adhered to contemporary national guidelines, indication bias may persist, possibly reflected in the differential distribution of high-risk factors between treatment groups. Although analyses have been adjusted for recognized prognostic factors, the possibility of preferential selection of more advanced cases for WPRT remains. Another factor that potentially complicate the interpretation of study results is the different time periods where the two treatment approaches were applied. Differences in treatment methods, such as the introduction of VMAT in 2014, as well as variations in patient selection criteria over time might result in imbalances between the treatment groups that are difficult to adjust for.

We observed a significant increase in acute GU toxicity and a numerical increase in cumulative late grade ≥ 3 GU toxicity with WPRT compared to PORT, possibly due to larger radiation volumes affecting bladder dose. Using a modified RTOG scale for toxicity grading, with lower criteria for grade ≥ 3 GU toxicity, might overestimate the severity of GU toxicity compared to other studies but should not affect within-study comparisons. Furthermore, image guidance based solely on bony anatomy is now considered outdated [25]. The larger PTV-margin and the inadequate image guidance could impact overall toxicity in both groups [26]. Previous evidence on WPRT-associated toxicity is conflicting [3], [5], [6], [9], [27], [28], [29]. The GETUG-01 trial [3] demonstrated no increase in GU or GI toxicity with WPRT. The RTOG 9413 trial [6] reported higher cumulative grade 3 GI toxicity with WPRT and neoadjuvant ADT. The POP-RT trial [27] initially showed an association between WPRT and late grade ≥ 2 GU toxicity, which diminished with longer follow-up [29]. The evidence on toxicity associated with hypo- and ultrahypofractionated WPRT is expanding, though the results remain variable [14], [30], [31], [32].

This study possesses notable strengths. The consecutive follow-up with prospective outcome registration, toxicity grading, and QoL assessment enhances data reliability. Meticulous verification of tumour characteristics and treatment-related variables minimizes information bias. The thorough evaluation of adverse effects and QoL parameters enhances the robustness of capturing treatment outcomes and patients’ experiences. Additionally, this study contributes valuable insights as one of the few exploring WPRT using moderate hypofractionation.

Despite its strengths, this study has limitations to consider. As discussed above, the two treatment approaches were applied in different time periods, complicating comparison due to variations in clinical practice over time. Moreover, discrepancies in the length of follow-up could potentially impact long-term outcome assessment reliability. The selection of BFFS as the primary endpoint may be subject to scrutiny, as MFS has been shown to be a more robust surrogate for OS [33], [34]. However, BFFS was chosen as the primary endpoint due to a larger number of events and the lack of regular radiological assessments, potentially limiting the reliability of MFS. Finally, toxicity grading was based on a locally modified RTOG-scale and only total-score for LiSat-11 was available. LiSat-11 has not been validated for assessing QoL following radiation therapy and lacks a defined Minimal Clinically Important Difference (MCID), making the clinical translation of differences between groups difficult.

In conclusion, the results of this study do not support a benefit of moderately hypofractionated WPRT over PORT in terms of survival outcomes but imply an increased risk of GU toxicity. Despite the limitations associated with study design, our findings reinforce the need for further studies investigating the clinical value of hypofractionated WPRT, considering a multilevel treatment assessment of efficacy, toxicity, and patient-reported outcome measures. Future results from ongoing trials, including RTOG 0924 and GETUG-AFU-23 (PEACE2), along with studies on hypofractionated WPRT such as PACE-NODES and HOPE, may provide valuable insights into the benefits and challenges of whole pelvis radiotherapy with varying treatment approaches.

## Source(s) of support

This study was supported by Region Örebro County, Sweden.

## CRediT authorship contribution statement

**Jenny Kahlmeter Brandell:** Conceptualization, Data curation, Formal analysis, Funding acquisition, Methodology, Project administration, Visualization, Writing – original draft. **Antonis Valachis:** Conceptualization, Methodology, Supervision, Visualization, Writing – review & editing. **Henrik Ugge:** Supervision, Writing – review & editing. **Daniel Smith:** Conceptualization, Formal analysis, Methodology, Supervision, Visualization, Writing – review & editing. **Bengt Johansson:** Conceptualization, Data curation, Investigation, Methodology, Supervision, Writing – review & editing.

## Declaration of Competing Interest

The authors declare that they have no known competing financial interests or personal relationships that could have appeared to influence the work reported in this paper.
