# Supplementary material for: Moderately hypofractionated prostate-only versus whole-pelvis radiotherapy for high-risk prostate cancer: A retrospective real-world single-center cohort study
Source: Clin Transl Radiat Oncol. 2024 Aug 21;48:100846. doi: 10.1016/j.ctro.2024.100846 (PMC11384977; doi:10.1016/j.ctro.2024.100846)
Supplement: Supplementary Data 1 [file mmc1.docx]

# Appendix A

## Table of Contents

[**Table A.1** Toxicity grading scale 2](#_Toc169022236)

[**Table A.2** Multivariate Fine-Gray regression analysis for metastasis free survival within the whole study population 3](#_Toc169022237)

[**Table A.3** Multivariate Fine-Gray regression analysis for prostate cancer specific survival within the whole study population 4](#_Toc169022238)

[**Figure A.1** Radiation volume per year of treatment 5](#_Toc169022239)

[**Figure A.2** Toxicity over time 6](#_Toc169022240)

[**References** 7](#_Toc169022241)

## **Table A.1** Toxicity grading scale

|  | **Grade 0** | **Grade 1** | **Grade 2** | **Grade 3** | **Grade 4** |
| --- | --- | --- | --- | --- | --- |
| **Genitourinary (GU)** | 0-1 nocturia, no dysuria or urgency | 2-3 nocturia, single dysuria or urgency | 3-4 nocturia, sporadic dysuria or urgency, or need of medical treatment | > 4 nocturia, severe urgency or need for temporary catheter | Need for permanent catheter |
| **Gastrointestinal (GI)** | 1-2 daily defecation, no urgency | 3-4 daily defecations, single urgency or bloody stool | > 4 daily defecations, frequent urgency or bloody stool, need of medication | Continuous problems or need for surgical treatment |  |
| **Erectile**  **Dysfunction (ED)** | Well-functioning erection | Weak or non-maintained erection | No erection |  |  |
| **Sexual life (SL)** | Satisfying sexual life | Impaired sexual life | No sexual life |  |  |

Toxicity grading scale, locally modified from Radiation Therapy Oncology Group (RTOG) grading scale [1].

## **Table A.2** Multivariate Fine-Gray regression analysis for metastasis free survival within the whole study population

| **Characteristic** | **HR***^1^* | **95% CI***^1^* | **p-value** |
| --- | --- | --- | --- |
| Radiation volume |  |  |  |
| PORT | — | — |  |
| WPRT | 1.49 | 0.81, 2.73 | 0.2 |
| Age | 0.99 | 0.95, 1.03 | 0.6 |
| Year of treatment | 1.11 | 1.01, 1.21 | 0.023 |
| T-stage |  |  |  |
| 1 | — | — |  |
| 2 | 3.23 | 1.46, 7.14 | 0.004 |
| 3 | 4.82 | 2.28, 10.2 | <0.001 |
| ISUP-grade |  |  |  |
| ISUP grade ≤ 3 | — | — |  |
| ISUP grade 4 | 1.76 | 1.05, 2.95 | 0.032 |
| ISUP grade 5 | 2.93 | 1.76, 4.89 | <0.001 |
| PSA | 1.00 | 1.00, 1.01 | 0.2 |
| Brachytherapy boost |  |  |  |
| No | — | — |  |
| Yes | 0.70 | 0.46, 1.06 | 0.10 |
| Hormonal therapy |  |  |  |
| None | — | — |  |
| Antiandrogen only | 1.17 | 0.34, 4.03 | 0.8 |
| ADT or CAB | 0.86 | 0.23, 3.24 | 0.8 |

^1^HR = Hazard Ratio, CI = Confidence Interval

Subdistribution hazard ratios for metastasis free survival, estimated using Fine-Gray regression, with death by any cause as competing risk event.

*Abbreviations:* PORT, Prostate Only Radiotherapy; WPRT, Whole Pelvis Radiation Therapy; ISUP, International Society of Urologic Pathologists; PSA, Prostate Specific Antigen; ADT, Androgen Deprivation Therapy; CAB, Combined Androgen Blockade, i.e., first generation antiandrogen in combination with Gonadotropin-releasing hormone-agonist or antagonist.

## **Table A.3** Multivariate Fine-Gray regression analysis for prostate cancer specific survival within the whole study population

| **Characteristic** | **HR***^1^* | **95% CI***^1^* | **p-value** |
| --- | --- | --- | --- |
| Radiation volume |  |  |  |
| PORT | — | — |  |
| WPRT | 3.09 | 1.23, 7.78 | 0.017 |
| Age | 1.04 | 0.97, 1.12 | 0.3 |
| Year of treatment | 1.10 | 0.96, 1.25 | 0.2 |
| T-stage |  |  |  |
| 1 | — | — |  |
| 2 | 6.10 | 1.40, 26.5 | 0.016 |
| 3 | 7.80 | 1.86, 32.8 | 0.005 |
| ISUP-grade |  |  |  |
| ISUP grade ≤ 3 | — | — |  |
| ISUP grade 4 | 1.59 | 0.77, 3.27 | 0.2 |
| ISUP grade 5 | 2.68 | 1.25, 5.76 | 0.011 |
| PSA | 0.99 | 0.97, 1.01 | 0.2 |
| Brachytherapy boost |  |  |  |
| No | — | — |  |
| Yes | 0.58 | 0.32, 1.04 | 0.068 |
| Hormonal therapy |  |  |  |
| None | — | — |  |
| Antiandrogen only | 1.03 | 0.37, 2.85 | >0.9 |
| ADT or CAB | 1.38 | 0.44, 4.32 | 0.6 |
|  | | | |

^1^HR = Hazard Ratio, CI = Confidence Interval

Subdistribution hazard ratios for prostate cancer specific survival, estimated using Fine-Gray regression, with death by other cause as competing risk event.

*Abbreviations:* PORT, Prostate Only Radiotherapy; WPRT, Whole Pelvis Radiation Therapy; ISUP, International Society of Urologic Pathologists; PSA, Prostate Specific Antigen; ADT, Androgen Deprivation Therapy; CAB, Combined Androgen Blockade, i.e., first generation antiandrogen in combination with Gonadotropin-releasing hormone-agonist or antagonist.

## **Figure A.1** Radiation volume per year of treatment

*
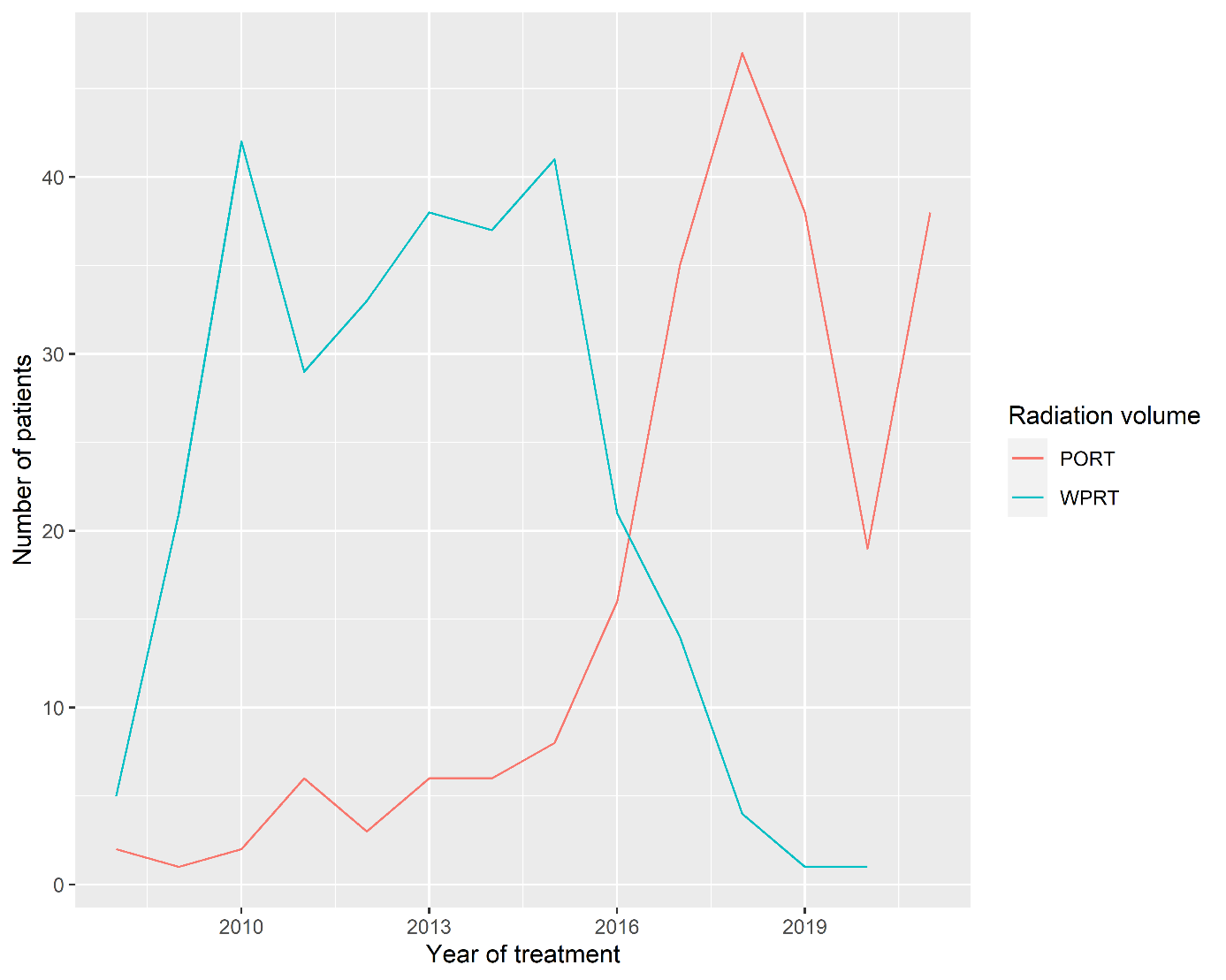
*

*Abbreviations:* PORT, prostate-only radiotherapy; WPRT, whole-pelvis radiation therapy.

## **Figure A.2** Toxicity over time


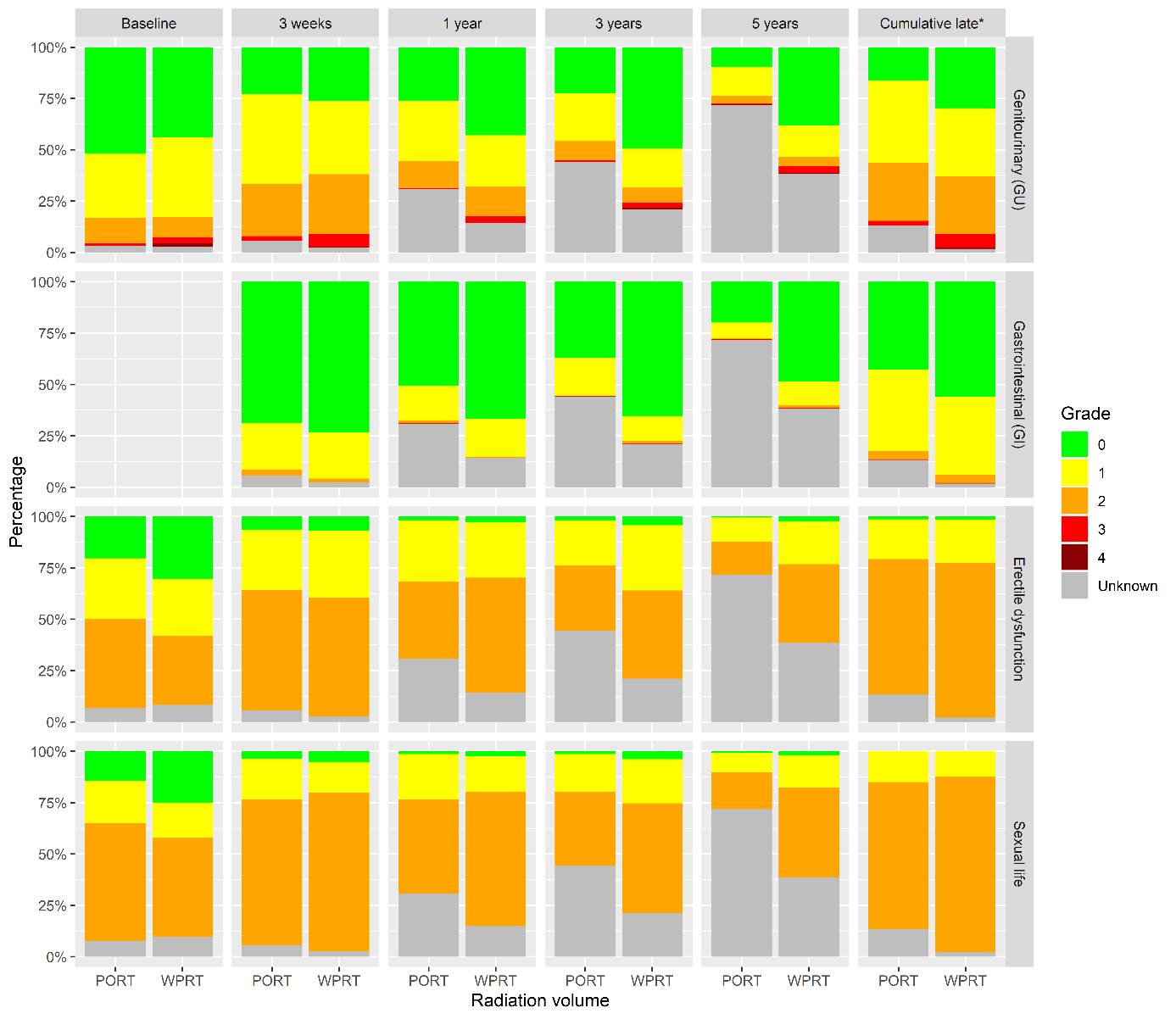


Toxicity grade distribution at baseline, three weeks, one year, three years, five years, and cumulative late toxicity (from six months to three years, measured every six months) for PORT versus WPRT. The timespan for cumulative late toxicity was set to three years to ensure that the selected time point would be shorter than the median follow-up in both treatment groups. Note that GU-toxicity was graded 0-4, GI-toxicity 0-3, ED and SL 0-2, according to table A.1.

*Abbreviations:* PORT, Prostate Only Radiotherapy; WPRT, Whole Pelvis Radiation Therapy; GU, genitourinary; GI, gastrointestinal; ED, erectile dysfunction; SL, sexual life

## **References**

[1] Cox JD, Stetz J, Pajak TF. Toxicity criteria of the Radiation Therapy Oncology Group (RTOG) and the European Organization for Research and Treatment of Cancer (EORTC). Int J Radiat Oncol Biol Phys. 1995;31(5):1341-6.
